# A survey of knowledge, attitudes, and practices towards skin and soft tissue infections in rural Alaska

**DOI:** 10.3402/ijch.v75.30603

**Published:** 2016-02-23

**Authors:** Gregory A. Raczniak, Joanna Gaines, Lisa R. Bulkow, Michael H. Kinzer, Thomas W. Hennessy, Joseph A. Klejka, Michael G. Bruce

**Affiliations:** 1 Arctic Investigations Program, Division of Preparedness and Emerging Infections, National Center for Emerging and Zoonotic Infectious Diseases, Centers for Disease Control and Prevention, Anchorage, AK, USA; 2 Epidemic Intelligence Service, Division of Applied Sciences, Scientific Education and Professional Development Program Office, Centers for Disease Control and Prevention, Atlanta, GA, USA; 3 Geographic Medicine and Health Promotion Branch, Division of Global Migration and Quarantine, National Center for Emerging and Zoonotic Infectious Diseases, Centers for Disease Control and Prevention, Atlanta, GA, USA; 4 Yukon-Kuskokwim Health Corporation, Bethel, AK, USA

**Keywords:** *Staphylococcus aureus*, MRSA, skin and soft tissue infections, Alaska Native people, traditional steambath, laundry practices, Maqiq, Aniinguaq

## Abstract

**Background:**

Community-acquired methicillin-resistant *Staphylococcus aureus* and methicillin-sensitive *S. aureus* infections are common to south-western Alaska and have been associated with traditional steambaths. More than a decade ago, recommendations were made to affected communities that included preventive skin care, cleaning methods for steambath surfaces, and the use of protective barriers while in steambaths to reduce the risk of *S. aureus* infection.

**Objective:**

A review of community medical data suggested that the number of skin infection clinical encounters has increased steadily over the last 3 years and we designed a public health investigation to seek root causes.

**Study design:**

Using a mixed methods approach with in-person surveys, a convenience sample (n=492) from 3 rural communities assessed the range of knowledge, attitudes and practices concerning skin infections, skin infection education messaging, prevention activities and home self-care of skin infections.

**Results:**

We described barriers to implementing previous recommendations and evaluated the acceptability of potential interventions. Prior public health messages appear to have been effective in reaching community members and appear to have been understood and accepted. We found no major misconceptions regarding what a boil was or how someone got one. Overall, respondents seemed concerned about boils as a health problem and reported that they were motivated to prevent boils. We identified current practices used to avoid skin infections, such as the disinfection of steambaths. We also identified barriers to engaging in protective behaviours, such as lack of access to laundry facilities.

**Conclusions:**

These findings can be used to help guide public health strategic planning and identify appropriate evidence-based interventions tailored to the specific needs of the region.

## Research Highlights

- Southwest Alaska Native people have up to 2 clinical encounters for skin infections per year.
- Prior public health educational messages for skin infections were understood and accepted.
- Communities believe skin infections are a problem and are motivated to prevent them.
- Interventions should include hand washing, clean water access, and surface barriers in steam baths.
- Novel foci were identified for future investigation to reduce skin infection burden.

Community-acquired methicillin-resistant *Staphylococcus aureus* (CA-MRSA) is an antibiotic-resistant form of the common bacteria *S. aureus* that occurs among persons outside of health care setting exposures or contacts. CA-MRSA and community-acquired methicillin-sensitive *S. aureus* (CA-MSSA) are spread by direct contact with infected surfaces and/or skin. This commonly presents as a skin and soft tissue infection (SSTI), which can become life threatening if not properly treated. SSTIs are common to southwestern Alaska (1–3). The Alaska Department of Health and the Arctic Investigations Program (AIP) of the Centers for Disease Control and Prevention (CDC) conducted an investigation in response to a rural Alaska community outbreak of *S. aureus* furunculosis in 1996 and showed that a quarter of residents reported having at least one boil during a 1-year period (3). Additionally, from March 1999 to July 2000, the total number of outpatient clinical visits to health care providers for skin infection complaints increased from 1 to 3.2% and the number of MRSA skin infection isolates increased from 5 to 56 per month (1). There were 240 culture-confirmed staphylococcal SSTIs, with 180 (75%) of these MRSA, and importantly, three-quarters of infections were found to be CA-MRSA. Illness was more common in persons who had received antimicrobial drugs during the year prior to the outbreak, who used crowded traditional saunas and whose household members had a recent history of furunculosis (3).

These reports led to the following recommendations and actions: 1) distributing standard responsible antibiotic treatment guidelines to decrease antimicrobial resistance to community health workers, 2) clean steambath seating areas after each use with dilute bleach solution, 3) use seating barriers during each steambath session, 4) limit the total number of persons using each steambath at one time, and 5) prevent persons with skin infections, sores, boils, furuncles, or carbuncles from using steambaths until the infection has stopped draining and healed (2–4). Over the past decade, regional hospitals conducted trainings and distributed information sheets to institute these recommendations.

Despite these actions, the rates of MRSA skin infections remain elevated among Alaska Native people ((5); Klejka J., personal communication, 2012). To assess previous educational programme effectiveness and identify additional interventions, we performed a study to capture the knowledge, attitudes and practices of rural Alaska Native people, using quantitative and qualitative components. Herein, we report results from a survey among persons residing in 3 south-western Alaska communities that had >0.6 skin infection clinic visits per person per year to determine: 1) knowledge of MRSA risk; 2) steambath hygiene practices; 3) preventive measures currently in use to reduce skin infections; 4) barriers to implementing previous recommendations; 5) community-generated solutions to prevent skin infections, and; 6) the acceptance and feasibility of interventions and education efforts to reduce skin infections. Stakeholders can use these data to develop evidence-based interventions aimed at reducing CA-MRSA and CA-MSSA in affected communities.

## Methods

### Investigation design

We used a cross-sectional mixed methods approach, collecting quantitative and qualitative data. Initially, we reviewed de-identified hospital and outpatient surveillance records for SSTI encounters (ICD-9 codes in the Appendix) provided by a southwestern regional hospital. From 1 January 2008 to 31 December 2011, the rate of clinical encounters per person per year was determined in 49 southwestern Alaska communities. We then designed a knowledge, attitudes and practices survey that was conducted in 3 communities (Communities A, B and C) that were found to have the highest rate of SSTI clinical encounters per person per year. The survey was administered via a 25-min face-to-face interview and consisted of 40 fixed-answer, quantitative questions and 28 open-ended qualitative questions at clinics or community-gathering centres. The principal investigator trained interviewers via mock interviews. Some terms in this report are specific to the Alaska Native people's community context and require definition. These include: washeterias are community facilities with the capacity for laundry and safe drinking water collection; self-haul water is collected from either a treated and purified source or a natural surface source and transported to the home; piped water is centrally treated in the community and piped directly to the home; steambaths are home-made plywood structures consisting of an entrance, antechamber and a steaming area that contains a space for sitting in front of a wood-fired metal boiler; “to steam” means to use a steambath.

### Ethics review, participant recruitment, and informed consent procedures

Three local communities (A, B and C) agreed to voluntarily participate in the study and additionally were the 3 communities with the highest SSTI clinic visit rate (per person per year). The CDC and Alaska Area Institutional Review Boards (IRBs) approved of this survey and determined this investigation to be a public health response activity under 45 CFR 46.102(d). We mobilized the community with posted flyers in the community's health clinic and stores, as well as short-wave radio announcements prior to and during our visit. Additionally, we recruited persons in the clinic waiting rooms, stores, and washeterias. Inclusion criteria included adult heads of households who were current community residents. We compensated each participant for their time with a $25 cheque. Prior to the survey, we obtained participant's verbal informed consent to ensure they understood the project and what participation involved. Consent forms were explained to the participant by investigation personnel; however, if required or desired, oral translation into local language by community health aides was provided.

### Data analysis methods, records management and participant confidentiality

Double data entry and analysis were conducted at AIP with STATA v10. Data were de-identified in order to keep confidential any specific information obtained during this investigation. For quantitative data, we used univariate and multivariable descriptive statistics. Qualitative data were analysed by a medical epidemiologist (GR) and a senior behavioural scientist (JG). After conducting a preliminary review of qualitative data, themes were developed and defined by consensus using the principles of grounded theory (6). Themes were then compared across questions and collapsed into meta-themes to more fully reflect the range of information gathered. These team members coded participant responses independently, and inter-coder reliability was acceptable (α≥90%).

## Results

### Descriptive epidemiology of clinical encounters for skin infections

**Fig. 1:**
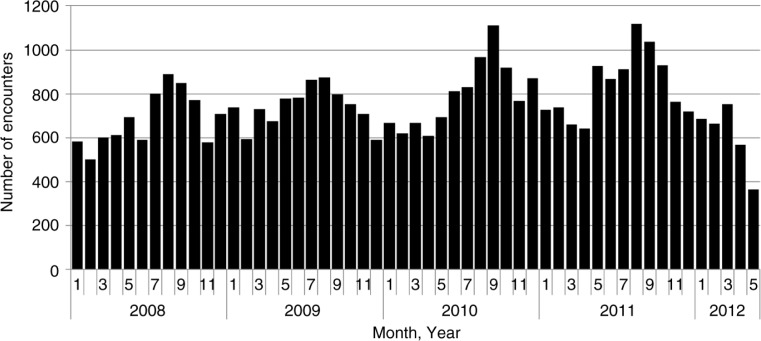
Skin and soft tissue infection clinic visits, all communities – southwestern Alaska, 1 January 2008–25 May 2012.

As shown in Fig. 1, peak incidence of hospitalizations and outpatient visits for SSTI encounters occurred in late summer and early fall (August–October). To determine in which communities to conduct this study, we determined the overall rate of clinic visits for SSTIs for the previous 4 years (Table I).

**Table I:** Skin and soft tissue infection clinic visit rate (per person per year) by communities of residence and year – south-western Alaska, 1 January 2008–31 December 2011^a^

Community	Total population in 2011, n	2008	2009	2010	2011	4-Year mean
A	418	1.65	1.83	2.11	2.23	1.95
B	590	1.04	1.17	1.41	1.00	1.15
C	721	0.79	1.10	0.88	0.63	0.85
D	576	0.61	0.67	0.61	0.60	0.62
E	498	0.81	0.76	0.61	0.29	0.61
F	275	0.41	0.63	0.52	0.72	0.57
G	462	0.74	0.38	0.44	0.52	0.52
H	655	0.22	0.39	0.60	0.80	0.51
I	1137	0.23	0.44	0.57	0.66	0.48
J	370	0.43	0.54	0.47	0.44	0.47
K	373	0.20	0.37	0.54	0.71	0.45
L	428	0.57	0.48	0.27	0.35	0.42
M	518	0.42	0.36	0.47	0.39	0.41
N	663	0.34	0.28	0.39	0.50	0.38
O	289	0.21	0.25	0.47	0.45	0.35

a Data shown are for the 15 communities in this region with the highest rates of skin infection visits.

### Knowledge, attitudes, and practice survey: quantitative findings

#### Demographic characteristics and community water sources

In these 3 communities, we administered interview surveys to 492 participants representing 48% of the total population over 18 years of age. This captured 77% of all households in the communities, which suggests that our sample is robust; however, a demographic comparison between participants and non-participants was not conducted. Women constituted 54% of volunteers and the average age of participants was 40.4 years (Table II). Respondents reported the water sources available in their community, as well as the water sources that they actually used (Table II). Community A does not have piped water, but does have a few homes with private wells as well as multiple public water stations close to homes. Respondents reported collecting as much rain water as possible for household use; the closest freshwater river was more than half a mile outside of town. Community B has piped running water to nearly every home, but not all residents could afford water service; thus, many residents haul their own water from treated or untreated sources. Community C does not have piped water and is located along a river. People reported that they found it easier to get river water available close to their homes despite treated water for purchase at the washeteria. Respondents in all communities indicated that they conserve water because it is either expensive and/or difficult to transport on a daily basis, especially in cold weather.

**Table II:** Participant demographics and reported sources of household water, by Alaska Native community

	A	B	C	Total
Population,^a^ n	418	590	721	1729
Population≥18 years of age^a^, n	257	335	434	1026
Number of participants^b^, n (eligible participating %)	102 (40%)	181 (54%)	209 (48%)	492 (48%)
Mean age of participants^b^, (years)	39.6	40.3	41.0	40.4
Male^b^ (%)	40%	48%	46%	46%
Number of households^b^	89	128	172	389
Households participating^b^, n (%)	60 (67%)	102 (80%)	138 (80%)	300 (77%)
Mean household size^b^	6.7	5.6	5.3	5.7
Piped water^b^ (% households)	2%	70%	6%	–
Self-haul treated^b^ (% households)	82%	29%	29%	–
Self-haul natural^b^ (% households)	93%	27%	94%	–

a US Census Data 2010

b This study 2012.

#### Knowledge and awareness of boils

Among the 492 study participants, 255 (52%) answered it was “very serious” to get a boil or “somewhat serious” (188, 39%). Forty-four respondents (9%) indicated boils were not a serious concern. Of the 391 that answered, 317 (81%) responded that boils were a problem in the community they lived in at the time of questioning. When asked a list of possible ways to get boils, 406/461 (88%) responded that sharing clothes or towels and 386/457 (85%) said touching other people's boils would be a way to get boils. About half of the people, 201/430 (47%), indicated that general body contact would be a way you can get a boil. When asked possible ways to prevent getting a boil, 420/478 (88%) reported that washing hands after touching items in public and washing clothing or bedding 426/475 (90%) were effective. Most people, 355/492 (72%), believed that taking a steambath with someone that has a boil would worry them and that they could get a boil if they did this. When queried “Are men or women more likely to get boils, or is their risk the same?” Out of 476, 104 (22%) felt men were more likely to get boils, while 358/476 (75%) felt the risk was the same and a minority 14/415 (3%) felt women were more likely to get boils.

We asked respondents if boils were a treatable disease and to list the best methods to cure boils. Among 469 that responded, 453 (97%) said boils could be cured, and that lancing the boil (233, 47%), washing (144, 29%), covering the boil with tape or bandages (128, 26%), and taking antibiotics (133, 27%) was curative. Just over 10% (52/492) of the people said that traditional methods were one of the best ways to cure boils. Few statistical differences between communities A, B and C were seen in these responses.

#### General hygiene practice

Because piped-in-home water service was not available in Community A, shower facilities were freely available only at the school. Over 70% of homes in Community B had showers and piped water service. Community C lacked in-home running water at the time of the survey, but there was a community shower available at the washeteria for a $3 fee. On average in Community C, people reported using a shower nearly once a week (0.83/week/person). Members of all 3 communities reported using traditional steam baths 3–4 times a week, on average. Our data show that the average person in community B (running water and easier access to showers) still preferred to use a steam bath, 2.7 times a week, compared to showering, 1.1 times a week (Table III). Communities without running water or washeteria showers nearly exclusively used steam baths for personal hygiene (Community A, Table III). Ninety-seven percent (480/492) of the respondents reported using an abrasive plastic wash cloth to clean their skin in steam baths, known locally as a “scrub” and 25% (117/492) reported that they shared these with other individuals. Everyone used a towel to dry and 14% (67/485) shared their towels with others (Table III). Cleaning of steam baths with bleach, Hexol or Pinesol at least one time per week was reported by respondents in 84% (185/221) of the households (Table IV).

**Table III:** Reported individual hygiene practices by community

	A	B	C	Total
Mean number of showers per person per week	0.3	1.1	0.8	0.8
Mean number of steam baths taken per person per week	3.7	2.7	2.7	2.9
Always use an abrasive scrub during steam, n (%)	101 (99%)	175 (97%)	204 (98%)	480 (97%)
Share scrub with others, n (%)	26 (25%)	40 (21%)	51 (25%)	117 (24%)
Always use a towel to dry off after steaming, n (%)	102 (100%)	179 (99%)	207 (98%)	488 (99%)
Share your towel with others, n (%)	13 (13%)	18 (9%)	36 (17%)	67 (14%)

**Table IV:** Reported frequency of cleaning the steambath, by household

	Community A	Community B	Community C
	
	50	67	103
<1 time per week (%, households)	2%	9%	28%
1–2 times per week (%, households)	36%	38%	40%
≥3 times per week (%, households)	63%	54%	33%

Women reported washing their hands (mean: 5.3 times per day) more frequently (P<0.001) than men (4.4 times per day). Overall, participants reported washing their hands 4.9 times a day, on average. In homes that lack running water, hand washing water may be collected in a basin and reused by multiple people; basin water was reported to be changed 3.3 times per day. Thus, for the average household size of our respondents (5.7 people), there were approximately 28 hand washing events a day, and if water is changed 3.3 times a day, we estimated that water was reused for 8.5 hand washing events.

**Fig. 2:**
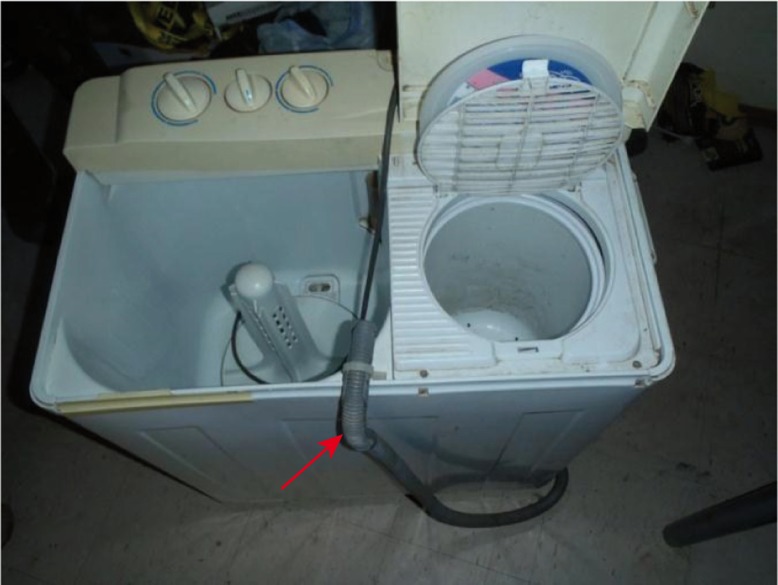
“Danby” clothes washing machine. The compartment on the left is used to agitate and clean clothes in water and detergent. The compartment on the right is a “spinner” that uses centripetal forces to remove water from clothes. You can see in this image that the water drainage tube (identified by red arrow) is re-feeding into the agitation compartment to “recycle” water for multiple loads of laundry.

Women reported doing laundry more frequently compared with men (64% vs. 20%) and also reported changing their clothes more often (4.6 times per week) than men (3.3 times per week). Significant differences were found in laundry rates when running water was available (2.8 versus 1.6 times per week, P<0.001). Those homes that use washing machines without plumbed water or piped drainage (“Danby” type, Fig. 2) reused laundry water for a mean of 3.1 laundry loads per water change as compared with laundry done in homes with running water (1.1 loads per water change, P<0.001).

#### Experience with boils

Of the respondents, 84% (415/492) reported having a boil in the past and 95% (466/492) reported knowing someone that had a boil. When asked about boils within the last 6 months, 17% (84/490) had a boil in the previous 6 months and 41% (200/492) had a household member with a boil in that time. Additional education programmes, if offered, was desired by 66% (324/492) but 57% (282/492) believed they knew enough about boils but thought others could use the education. When asked how to get this information to them and their communities, 49% wanted printed flyers or brochures sent to their homes, 29% wanted to get the information from health aides and 13% wanted the information from radio, newspaper or community meetings.

#### Willingness to change practices

We asked people if they would be willing to change personal habits if it would decrease the risk of boils. Of 490 respondents, 278 (57%) indicated they would be willing to make changes. However, when asked about specific items, 337 of 491 (69%) said they would be willing to try a disposable towel impregnated with soap or antiseptic media, 428 of 490 (87%) would be willing to use hand sanitizer, 387 of 449 (86%) said they would sit on a barrier in the steam bath, 443 of 488 (91%) said educational programmes for children would be helpful, 415 of 488 (85%) said educational programmes for adults should be provided, 327 of 490 (67%) would be willing to change clothing more frequently, 332 of 485 (68%) would wash clothing more frequently, 417 of 492 (85%) would clean their counters and toilets 3 or more times a week and 379 of 468 (81%) were willing to clean their steam baths after every use. Of those who indicated that barriers in the steam would be a good idea, many people said they already engage in this practice. When asked what they use, 113 of 148 (76%) responded that they sit on cloths or towels. When asked what would be the ideal material for a steam barrier, 243 (49%) wanted to use cloths or towels, 76 (15%) said cardboard or paper, 41 (8%) said plastic, 38 (8%) said wood, and 40 (8%) said other materials, such as rubber.

### Knowledge, attitudes, and practice survey: qualitative findings

#### General knowledge, attitudes and practices

Several respondents, when asked “What is a boil?” indicated some knowledge of boils and had ideas about what a boil is, what are the symptoms of a boil, and how to prevent boils. No respondent indicated any positive associations with boils; when asked “How do you feel when you have a boil?”, several indicated a sense of stigma or shame associated with having them. When asked, “What would you do if you thought you had symptoms of a boil?”, multiple individuals did recognize the potential severity of boils as a medical problem, most respondents indicated that they believed boils did not warrant formal treatment by a trained medical professional, and except if previous treatment at home had failed. Many respondents offered descriptions of boils when asked, “What is a boil?” that reflected understanding boils as an infection. These included mentions of *bacteria*, *staph*, *sepsis*, *celluliti*, *pus* and *MRSA*. Boils were described as being physically painful and even potentially dangerous: “it might go to my vein.”

When asked what worried them the most about boils, respondents typically cited inadequate water and sanitation, physical or emotional pain, and concerns about the infection worsening or spreading. Water and sanitation were specifically referenced as a lack of running water causing boils or sewage spreading the disease among community members.

Respondents identified steam bath use and/or general sanitation/hygiene practices as the main risk factors for boils. Several respondents also identified steaming with an individual who had boils at the time as a possible risk factor. Multiple respondents also indicated that individuals who steamed more often were more likely to get boils. Dirty clothes and steam baths were cited as possible sources of boils, as were inadequate hand washing and dirty fingernails. Although it was not common, some respondents indicated that they felt boils were idiopathic: “it just happens.” Of note, multiple participants expressed the idea that boils could be caused by some type of break in the skin, such as by scratching or a mosquito bite, and it was this opening that allowed boils to grow.

When asked why a respondent believed men were more likely to get boils than women, this was most often explained to be a result of their jobs. Men were described as having occupations that exposed them to dirt and being responsible for household chores such as emptying the “honey bucket” (a plastic 5-gallon bucket used to collect faeces and urine) and going hunting. Other reasons that men were more likely to get boils included: scratching more often, changing clothes less frequently than women, steaming at a higher temperature and more frequently with a larger group of men, and having an immune system that was fatigued and unable to fight off infection.

#### Boils treatment

When asked how to treat boils, the majority of respondents indicated that boils could be successfully treated at home. Traditional healing methods included covering a boil with tape and using some type of hot pack. Herbs such as fireweed, stinkweed or tobacco were reportedly used to treat boils, and tundra moss was used as a wound cover as well to aid the healing process. Several respondents reported using kerosene, alcohol, or body spray containing alcohol to treat boils. Many respondents did indicate that boils could be cured through treatment at a clinic, but this was identified as a resource to be used only after previous attempts at treatment had failed. Three themes were identified for not pursuing medical treatment for boils: lack of perceived severity, perceived manageability at home and negative associations with medical care. Some participants reported negative associations with medical care which typically reflected a previous experience the respondent had with formal treatment indicating fear or dismissal: “Health care worker laughed at me [the] first time I came with a boil. I just have other people help me now.” Several respondents reported that one could cure and/or prevent boils through eating the pus or head of a boil. The practice was explained as a way that the body could become familiar with the bacteria. One participant even said, “[The] body will know what to fight like a vaccine booster shot.”

#### Boils prevention

The importance of hygiene was identified as a common theme in boils prevention. Hygienic practices such as laundering clothes, bathing, washing hands and disinfecting objects were the most common specific practices cited by respondents. The most commonly identified effective cleaning solutions were specific products like bleach, Clorox, Hexol, or Pinesol or hot and soapy water. Wearing clean clothes, using clean towels and keeping a clean house were all identified as ways to prevent boils. Multiple respondents identified sitting on rags or towels in the steam bath as an effective method of preventing boils. Another effective behaviour to prevent boils that was identified was avoiding scratching boils to prevent their spread. This is similar to the identified belief that boils can be caused by a break in the skin.

#### Steam bath use

When asked, “Why do you steam?” participants most commonly identified personal hygiene and cultural practices as the reason. Individuals who used steam baths for hygienic purposes described using them in order to “sweat out my germs”; several participants identified steaming as making an individual feel cleaner than taking a shower would. Steaming was also described as being adequate for personal hygiene if there was no access to running water.

As a social and cultural practice, steaming offers residents an opportunity to visit with others. Steaming was also described as being physically and psychologically soothing for participants. Individuals reported that while they knew steaming with someone who had a boil put them at risk for a boil, they did not always feel comfortable asking someone with a boil to abstain from using the steam bath or that they did not want to stigmatize another community member. Others indicated they would feel comfortable steaming with a partner who had a boil because they felt that the risk was low or that they could protect themselves through using separate towels or bandaging the boil to prevent exposure.

#### Steam bath disinfection

Responses about steam bath cleaning included a reference to specific disinfection products like Pinesol or Clorox. Participants also frequently claimed that they currently cleaned their steam bath either before or after use. Barriers to regular cleaning of the steam bath included: lack of running water, expensive or unavailable disinfection products, and the significant amount of effort associated with cleaning the steam bath at such a high frequency. Regular cleaning of the steam bath was also identified as necessary only if they believed the steam was contaminated (e.g. through use of the steam by someone with a boil).

## Discussion

In this investigation, we evaluated the knowledge, attitudes and practices related to skin infections and boils among residents of 3 south-west Alaska communities, where the rate of skin infections is high. The strengths of this project were the use of quantitative and qualitative questions, the high response rate and the variability in access to in-home water services among the residents. This allowed us to gather in-depth information that reliably reflects these communities and allows us to contrast the practices used based on access to water services. Although several investigations have described the epidemiology of SSTIs in Alaska, a mixed quantitative–qualitative approach has not been previously attempted.

We found no prevalent misconceptions surrounding the aetiology and pathology of boils that would be a barrier to further prevention or education efforts to prevent boils. Previous public health messages disseminated to the inhabitants of the communities studied were understood and generally accepted. Respondents were concerned about boils as a health problem and motivated to prevent boils. Many effective prevention and treatment behaviours were observed and reported; however, we identified some practices that could be improved and barriers that could prevent people from protecting themselves from developing boils. These practices included: inadequate hand washing due to reuse of water and limited access to soap; laundering clothes with water used for multiple washloads; not using heat to dry clothes after laundering (both a lack of access with only a few dryers in washeterias and lack of resources to pay to use the dryers); frequent skin contact with potentially contaminated surfaces such as steam baths; and the sharing of scrubs and towels.

Our data suggest that previous efforts to educate community members on the importance of cleaning steam baths were successful. Steam baths have been identified as a risk factor for boils in this region of Alaska, and they are commonly used in these communities (1,7). Health messages that emphasize avoiding steam baths when an individual has a boil, including information on alternative social activities to steaming when one has a boil, may decrease exposure and transmission.

Boils were commonly understood to be a skin infection caused by *germs*, and descriptions of boil transmission as a result of breaks in the skin suggest an understanding of transmission consistent with established biologic mechanisms of how boils are transmitted. The qualitative data finding that persons identified breaks in the skin as an important route of transmission for boils complements quantitative data findings regarding the use of scrubs during steam bath routines. Idiopathic modes of transmission were rarely identified by respondents. Furthermore, there was a general sense that other people's steam baths may be unclean, but no one ever said their own steam bath is not clean. Additionally, while many respondents said they clean their steam baths, there is little household accountability to ensure adequate steam bath hygiene. Educational messages should address the potential for scrubs to abrade users’ skin. Also, there seemed to be awareness among respondents of “scratching with dirty nails,” or even dirty hands as a risk factor. This supports the idea that the message that “germs cannot be seen” should be reinforced. Importantly, cost for health care was not identified as a barrier, perhaps because traditional methods are often tried, or because clinic care is available with governmental prepaid health care within the community. This suggests that underreporting of skin infections to clinical providers is highly likely. Home treatments could also contribute to high rates of complicated boils and increased intra-household transmission via inadequate incision and drainage, poor infection control and insufficient wound care. Initial self-treatment has also been described in Asian/Pacific Islanders (A/PIs), but this group differed from Alaska Native people in that A/PI respondents did not recognize this infection as potentially severe (8).

Taken together, these findings suggest that there is a need for educational materials focussing on the transmission of skin infections among household members, as well as emphasizing which individuals are most at risk for skin infections. Materials should also emphasize that people can spread germs within their own steam baths, germs can live in a steam bath for a long time and floors do not have to be *slimy* to be contaminated. Thematically, it is difficult to separate the act of steaming as a risk factor versus steaming in an unclean/unsafe way as a risk factor.

Special attention should be paid to the development and implementation of educational efforts. In northern Saskatchewan, epidemiologists found that following the initiation of an educational programme that developed physician, patient, community and school-based educational materials, the rates of MRSA infections decreased twofold. Through pre- and post-educational intervention surveys, the researchers demonstrated that the decrease in MRSA infections coincided with a community-wide increase in hand washing and knowledge related to antibiotic use (9,10). However, many respondents in our cohort indicated that adults were already sufficiently aware of the problem of boils and expressed doubt that additional educational programmes would be effective. Additionally, a high proportion of northern Saskatchewan's population has access to piped treated water systems, which perhaps made this educational messaging more effective. These findings suggest that the communities studied may be experiencing fatigue as a result of previous efforts to decrease SSTIs. Prior to community-wide implementation, piloting these educational materials and lectures would be beneficial to ensure appropriateness and acceptance.

Our investigation had several limitations. All data were collected through self-report. While our findings generally indicate that knowledge was high, one participant admitted, “I don't always follow what I'm going to say …” We did not perform household visits which could have established the presence of appropriate disinfection products used for steam baths and would have offered a more objective measurement of household hygiene practices. Another objective measure that could be used would be sales records of disinfection products at the local store to determine if there were any changes in the amount of cleaning products being purchased. Another potential limitation is the lack of behaviour change assessment; a future study might include an ethnographic component in which household visits would assess not only the presence of disinfection products but also their appropriate use. We visited a limited number of communities which may not be representative for the entire region. Also, we only visited at one point in time; therefore, if attitudes and practices change with the season, then our data are temporally limited.

Participants were not able to actually experience any of the proposed materials or interventions in this setting and therefore findings are limited to the participant's impressions of such interventions. Respondents relied on previous experiences to form an idea of what these materials might look like or how they would actually work or be used. So, individuals may have had different concepts of what the interventions would be. Confusion about the proposed interventions was also evident in several responses, such as when an individual identified a “disposable soap towel” as being something that would only be used for travelling or only on their hands. Social desirability bias could have also affected respondents as participants may have felt that endorsing different materials would result in their receipt of an item free of charge or the promise of future services or assistance. We hope that this initial report can guide future interventions.

## Conclusions

We want education to be grounded in theory and evaluated for efficacy, and educational materials to fit the unique culture and socioeconomic reality of this population. Using the Health Belief Model's constructs, we recommend a focus on the following: 1) Perceived susceptibility: Education materials should include information on risk factors for boils; 2) Perceived severity: Provide instruction that boils can spread, that there is no acquired immunity, how to recognize the signs/symptoms of a boil before they become more serious conditions, and that *S. aureus* infections can be serious; 3) Perceived barriers: Information on addressing perceived barriers (e.g. the availability of effective disinfection products, the challenges of regular cleaning and the availability of alternatives to bathing in steam baths); 4) Perceived benefits: What “success” looks like (e.g. cleaner households, fewer skin infections, less absenteeism and fewer clinic visits). We also believe other theoretical concepts should be incorporated into educational materials, like social normalization which encourages proactive behaviour, specifically involving traditional leaders in the message development and dissemination to increase their acceptability and implementation.

## Appendix: ICD9 codes used in this investigation

**Table:**

680	Carbuncle and furuncle
680.0	Boil, face
680.1	Boil, neck
680.2	Boil, trunk
680.3	Boil, arm
680.4	Boil, hand
680.5	Boil, buttock
680.6	Boil. leg
680.7	Boil, foot
680.8	Boil, site NEC
680.9	Boil, NOS
681	Cellulitis and abscess of finger and toe
681.01	Felon
681.02	Paronychia, finger
681.1	Cellulitis and abscess of toe
681.11	Paronychia, toe
681.9	Cellulitis/abscess, unspec. Digit
682	Other cellulitis and abscess
682.0	Cellulitis/abscess, face
682.1	Cellulitis/abscess, neck
682.2	Cellulitis/abscess, trunk
682.3	Cellulitis/abscess, upper arm
682.4	Cellulitis/abscess, hand
682.5	Cellulitis/abscess, buttock
682.6	Cellulitis/abscess, leg
682.7	Cellulitis/abscess, foot
682.8	Cellulitis/abscess, NEC
682.9	Cellulitis/abscess, unspec
V09.0	Infection with microorganisms resistant to penicillins
V02.53	Carrier or suspected carrier of Methicillin susceptible *Staphylococcus aureus*
V02.54	Carrier or suspected carrier of Methicillin resistant *Staphylococcus aureus*
V12.04	Personal history of Methicillin resistant *Staphylococcus aureus*
038.11	Methicillin susceptible *Staphylococcus aureus* septicemia
038.12	Methicillin resistant *Staphylococcus aureus* septicemia
482.41	Methicillin susceptible pneumonia due to *Staphylococcus aureus*
482.42	Methicillin resistant pneumonia due to *Staphylococcus aureus*
041.11	Methicillin susceptible *Staphylococcus aureus* in conditions classified elsewhere and of unspecified site
041.12	Methicillin resistant *Staphylococcus aureus* in conditions classified elsewhere and of unspecified site
